# Effectiveness of a cognitive behavioural therapy-based anxiety prevention programme at an elementary school in Japan: a quasi-experimental study

**DOI:** 10.1186/s13034-018-0240-5

**Published:** 2018-06-19

**Authors:** Yuko Urao, Michiko Yoshida, Takako Koshiba, Yasunori Sato, Shin-ichi Ishikawa, Eiji Shimizu

**Affiliations:** 1 Research Centre for Child Mental Development, Chiba University Graduate School of Medicine, 1-8-1 Inohana, Chuo-ku, Chiba, 260-8670 Japan; 2 Department of Cognitive Behavioural Physiology, Chiba University Graduate School of Medicine, 1-8-1 Inohana, Chuo-ku, Chiba, 260-8670 Japan; 3 Department of Global Clinical Research, Chiba University Graduate School of Medicine, 1-8-1 Inohana, Chuo-ku, Chiba, 260-8670 Japan; 4 Department of Psychology, Doshisha University, 1-3 Tatara Miyakodani, Kyotanabe, Kyoto 610-0394 Japan; 5 Department of International Communication, Kanda University of International Studies, 1-4-1 Wakaba Mihama-ku, Chiba, 261-0014 Japan

**Keywords:** Anxiety, Prevention, Cognitive behavioural therapy, Elementary school, Universal, Japan

## Abstract

**Background:**

The efficacy of cognitive behavioural therapy (CBT) for anxiety related problems in children is empirically supported. In addition, universal anxiety prevention programmes based on CBT have been demonstrated in recent years. The purpose of this study was to verify the effectiveness of a CBT based original programme ‘Journey of the Brave,’ aiming to prevent anxiety disorders and anxiety-related problems for Japanese children aged 10–12 years old.

**Methods:**

Intervention groups from two classes of 5th grade elementary students (*n* = 41) received ten 45-min programme sessions. The control group was drawn from one class of 5th grade children (*n* = 31) from a nearby school. All participants completed the Spence Children’s Anxiety Scale (SCAS) at pre, post, and 3 months follow-up. Mixed-effects model for repeated measures analysis was conducted.

**Results:**

The mean anxiety score on the SCAS for the intervention group was significantly reduced at both post intervention and 3 months follow-up compared with the control group. The group differences on the SCAS from baseline to post-test were − 5.321 (95% CI − 10.12 to − 0.523, *p* = 0.030), and at the 3-month follow-up were − 7.104 (95% CI − 11.90 to − 2.306, *p* = 0.004).

**Conclusions:**

The effectiveness of the anxiety prevention programme ‘Journey of the Brave’ was verified though this study using a quasi-experimental design on a small sample.

*Trial registration*: UMIN000009021

## Background

Anxiety disorders are the most common mental health problem in children and adolescents [1] with lifetime prevalence rates averaging between 8 and 27% [2]. Despite the high prevalence rates, many children and adolescents remain undiagnosed and untreated [3].

Children and adolescents with anxiety symptoms and disorders suffer from considerable adverse effects on their psychosocial functioning [4]. For example, many studies report that anxiety symptoms in childhood significantly interfere with children’s interpersonal functioning, self-esteem, social competencies, and academic achievement [1, 5, 6]. Moreover, comorbidity between anxiety and other psychological disorders is common, and there is evidence suggesting that anxiety disorders may precede the onset of other psychological disorders including depression [7–9].

Because of the high prevalence rates and considerable adverse effects of anxiety symptoms and disorders, the need to prevent anxiety disorders in children is paramount. The application of prevention science to reduce anxiety disorders, however, is in its infancy [10].

There are three approaches to addressing mental disorders of children: (1) universal approach, (2) selective approach, and (3) indicated approach [11]. The universal approach works with all children, including those who have no disorder symptoms, while the selective approach targets children with specific risks and the indicated approach is for those with subclinical signs or symptoms [12]. In 2000, Offord summarized the advantages of universal programmes as avoiding participants’ stigma, such as the labelling effects of being singled out, and reaching those with a wide range of risk factors rather than those with a limited number of factors [13].

Studies attempting to verify the effectiveness of anxiety prevention programmes for children have been gradually increasing since 2000. Several studies have systematically reviewed the effectiveness of various preventive programmes and suggested that many universal, selected, and indicated approach programmes demonstrate effectiveness with small to medium effect sizes [3, 4, 14, 15].

Cognitive behavioural therapy (CBT) is a psychological treatment method which has been verified to be effective against anxiety disorders in children in many studies in both individual [16, 17] and group formats [18, 19]. Currently, it is suggested that CBT is one of the evidence-based treatments for anxiety disorders in children [20, 21]. The FRIENDS programme has been verified to be effective not only for treatment of children with anxiety disorders but also as a preventive intervention. FRIENDS was originally developed by modifying the Coping Cat programme [22] which was developed by Kendall in the USA for the treatment of children’s anxiety disorders. The effectiveness of the FRIENDS programme conducted with the universal approach has been verified in various random block design studies from the early 2000’s [23–26]. Effectiveness trials of the FRIENDS programme have been conducted in several countries and are ongoing [27–29]. Moreover, a new trend is emerging. Specifically, country-adapted programs which are designed to be suitable for children in specific countries are being developed and examined. For example, Collins and colleagues [30] studied the effects of a universal school-based mental health intervention in schools in Scotland and reported significant anxiety reduction and improved coping at post-intervention and again at a 6-month follow-up compared with a comparison group who received regular personal and social education (PSE) sessions [30]. In a randomized controlled trial, Calear and colleagues in Australia randomly assigned children from three schools to intervention and control groups, and although children in the intervention group only undertook an online CBT based anxiety prevention programme, there were no significant score differences between groups on anxiety, depression, and other measures of mental wellbeing at post programme and follow-up timepoints [31].

While evidence of the effectiveness of CBT based anxiety prevention programmes has been compiled overseas, the progress of research and implementation is very slow and there is little mental health education targeting anxiety prevention in Japan. In this country, anxiety issues are handled in the mental health sessions of Physical Education classes of 5th grade elementary school children with remarks such as ‘In order to deal with anxiety issues, there are options such as to consult, to play, and to exercise’. Furthermore, only three 45-min sessions of class time are allocated [32] and there is no systematic education based on psychiatry or psychology.

Under these circumstances, adapting an anxiety prevention programme proven effective in western countries to Japanese school classes is promising. However, there are many potential obstacles such as time requirements (ten sessions plus a booster session of 60–120 min/session), personnel and financial costs (teacher training and workbook), and introduction methods (group work format, parent/facilitator participation desired, etc.). It has been pointed out that ‘the programme which turned out to be effective in preceding studies in other countries is not always successful in this country’ [33]. Therefore, it is necessary to develop an adapted programme and examine its effectiveness in Japanese schools.

We developed a new CBT based anxiety disorder prevention programme, ‘Journey of the Brave,’ for Japanese children [34]. The pilot study indicated a significant improvement in anxiety scores on parents’ evaluation at 3 months compared with the control group, although there was no significant improvement in children’s reports. In addition, equal or better effect sizes were found in both children’s (*d* = 0.72) and parents’ (*d* = 0.65) ratings in comparison with the sizes shown by previous research [3, 14]. Therefore, effectiveness and feasibility of the ‘Journey of the Brave’ programme were partially demonstrated, although its feasibility and effectiveness should be addressed in more robust future research, especially in regular Japanese school classrooms. As such, this study examined the effectiveness of the programme for anxiety symptoms in children when implemented in a regular school class as part of the school curriculum.

## Methods

### Study design and participants

This was a universal quasi-experimental study with an intervention and a control group. Intervention group participants received the anxiety prevention programme and control group participants received no intervention.

The intervention group consisted of 41 5th grade children (10–11 years old, two classes) attending an elementary school in City A adjacent to Tokyo. The control group consisted of one class with 31 children of the same age at another nearby elementary school in the same city. The control group school was selected based on the nomination from the principal of the intervention group school.

The research design for this study involved measuring children’s anxiety levels across three time points during the school year. Data were collected at pre-test (Time 1; week 0), post-test (Time 2; month 6), and follow-up (FU; Time 3; 3-months following post-test).

### Intervention—Journey of the Brave

The following is a summary of the ‘Journey of the Brave’ programme.

This programme consists of ten 45-min sessions and the contents are taught according to a workbook and teacher’s manual (Table 1). The first half of the programme is dedicated to the development of anxiety stairs and the experience of exposure, while the second half mainly concerns cognitive restructuring. More precisely, after psychological education regarding anxious feelings (i.e., anxiety is a natural feeling everybody has and plays an important role in protecting you from danger, but if excessive anxiety persists, it may lead to disturbances in life, etc.), each child is encouraged to establish his or her own goal throughout the program such as to make a presentation in front of all the children, to remain alone in a dark room, and so on. In stage 3, relaxation skills such as breathing methods and muscle relaxation are taught. In stage 4, children develop the anxiety stairs table to reach the goal set in stage 2. Stages 5, 6, and 7 encompass the process of gradually learning the cognitive model (the relationship between cognition, behaviour, emotion, and body reaction) as well as cognitive restructuring. At the same time, gradual exposure homework to address anxiety proceeds in accordance with the anxiety stairs table developed in stage 4. Assertion skills to reduce interpersonal anxiety are taught in stage 8, stage 9 is the overall review session, and stage 10 is the summary and graduation ceremony.

**Table 1:** Contents of ‘Journey of the Brave’ by session

Session	Content of Journey of the Brave
1	Understanding of four basic feelings
2	Monitoring feelings of anxiety and setting goals
3	Body reactions and relaxation
4	Anxiety level stages and stair step exposure
5	Anxiety cognition model
6	Identify cognitive distortions and coping with rumination
7	Cognitive restructuring when anxious
8	Assertiveness skills to reduce social stress
9	Review
10	Summary

In the workbook used by the children, actual examples of many anxiety-provoking moments in their daily life are raised so that they can deepen their understanding of anxious feelings and CBT.

### Procedure

The 45-min ‘Journey of the Brave’ programme sessions were conducted in the intervention group over 6 months from October 2013 to March 2014 at a pace of twice a month, although no sessions were held during winter holidays.

Each session started with a PowerPoint presentation and workbook and homework sheets were distributed. Homework to consolidate the content learned was given at the end of each session to be worked on at home and returned by the next session.

The actual intervention was conducted by TK who is an expert (MA) in education psychology. She met with the programme author YU prior to each session to discuss facilitation. In addition, YU supported the facilitator in the class during the session and the teacher in charge of the class was also present providing partial support.

Children in the control group followed the regular school curriculum led by the classroom teacher.

Each of the 45-min sessions were conducted with the intervention group children in regular classes. The programme contents were supervised by a MD/PhD university professor who is a CBT expert.

### Measurements

#### Primary-outcome measure

##### SCAS: Spence Children’s Anxiety Scale

The primary outcome measure was the Spence Children’s Anxiety Scale (SCAS) which is one of the most valid self-reported measures for assessing child anxiety meeting diagnostic standards for 8- to 15-year-old children [35]. Reliability and validity of the SCAS Japanese version has been confirmed [36]. The SCAS includes 38 items regarding children’s anxiety symptoms divided into six subcategories: separation anxiety, social phobia, panic disorder/agoraphobia, generalized anxiety disorder, physical injury fears, and obsessive–compulsive disorder. The SCAS item scores range between 0 (never) and 3 (always) and the maximum possible score is 114. According to a previous study, the average SCAS score of 7 to 12-year old children was 20.51 (SD = 14.20) and the cut-off point was 42 [37].

Furthermore, one additional question was added as the 39th question—‘Are any of the items severely negatively affecting your daily life?’—to be answered in the same 0–3 scale to evaluate the degree of severity of the anxiety symptoms affecting the child’s daily life.

#### Secondary-outcome measure

##### SDQ: Strengths and Difficulties Questionnaire

The secondary outcome measure was the self-report version of the Goodman Strengths and Difficulties Questionnaire (SDQ) [38]. The SDQ includes 25 items, each scored 0 (not true), 1 (somewhat true), or 2 (certainly true) according to the perceived severity of the symptom. The items are divided into five subcategories: emotional symptoms, behaviour problems, hyperactivity/inattention, peer relationship problems, and pro-social behaviour. A total difficulty score is computed by summing scores of the first four sub categories and the maximum possible score is 40.

### Statistical analysis

The statistical analysis and reporting of this trial were conducted in accordance with the CONSORT guidelines, with the primary analyses based on the intent-to-treat principle. For baseline variables, summary statistics were constructed using frequencies and proportions for categorical data and means and SDs for continuous variables.

The participant characteristics were compared using Chi squared tests for gender differences and t tests for baseline score differences between the intervention and control group.

Primary analysis was performed with the mixed-effects model for repeated measures (MMRM) with treatment group, time, and interactions between treatment group and time as fixed effects; an unstructured covariate was used to model the covariance of within-subject variability. MMRM analysis assumes that any missing data occur randomly. The secondary analysis was performed in the same manner as the primary analysis. All comparisons were planned and all p-values were two-sided. A p-value < 0.05 was considered statistically significant. All statistical analyses were performed using the SAS software program, version 9.4 (SAS Institute, Cary, NC, U.S.A.) and SPSS Version 22.0 (IBM, Armonk, New York, USA).

## Results

Gender-based differences in participant characteristics were examined with Chi square tests between the 41 intervention group (male = 21, female = 20) and 31 control group (male = 16, female = 15) children at pre-test. There were no significant differences (*p* = 0.974). Next, to compare the group differences at baseline, t tests were conducted of the pre-test SCAS and SDQ scores. The intervention group demonstrated significantly higher scores on the two measures than the control group (SCAS: *p* < 0.01, SDQ: *p* < 0.01).

**Fig. 1:**
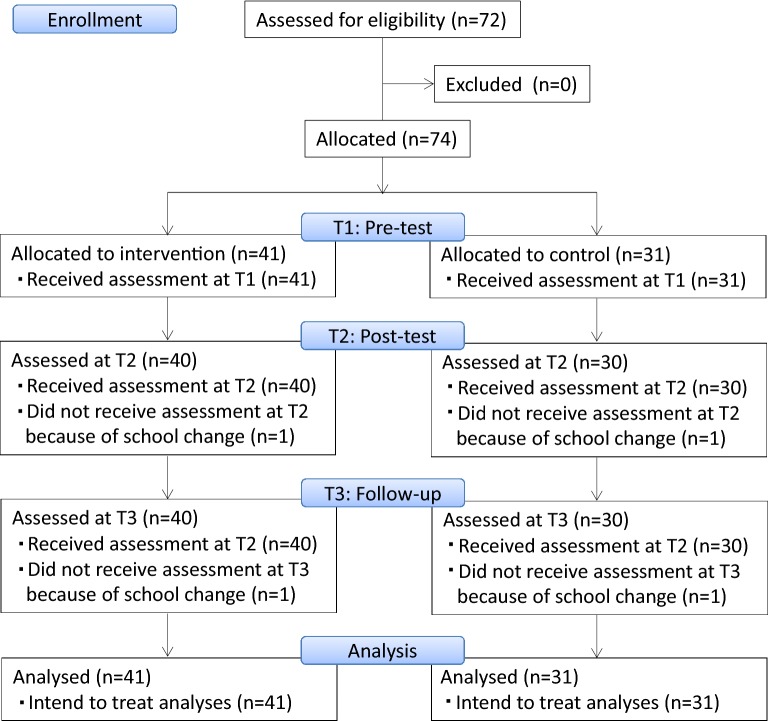
Flow-chart. It displays the number of children at each time point and a sample count of ITT analysis. *ITT* intention to treat

All ten sessions were held in the classroom of the intervention group during regular school class time. There were no dropouts since all the children who were present participated in the programme. Self-reported questionnaires were distributed to the children from the teacher in charge of each class and all the children in the class (41 intervention group and 31 control group) answered. In the process, the teachers assisted the children by orally reading out the questions. The data count was reduced only by one at post-test and at follow up because one child from each group left school (Fig. 1).

**Fig. 2:**
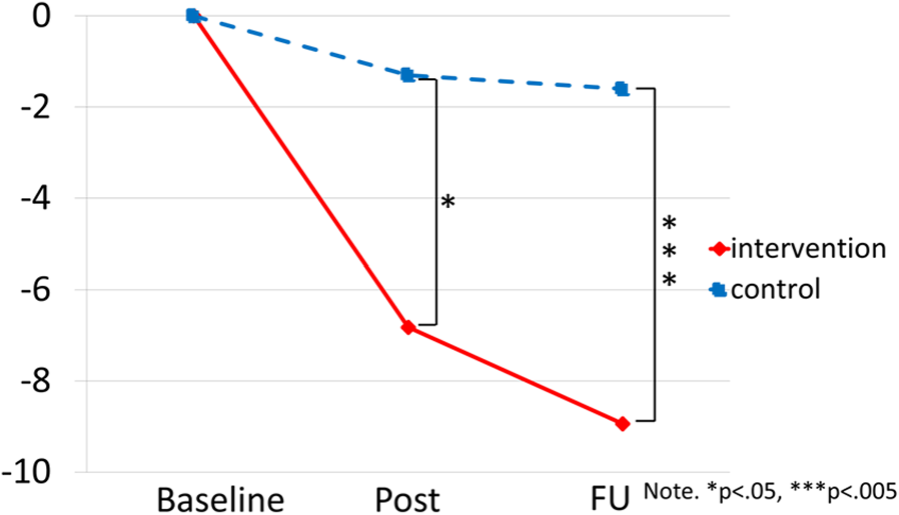
Mean total SCAS scores in each group during the study. It displays the average SCAS scores of the intervention group and the control group for each time point

After 6 months (the post-test), the adjusted means of the SCAS were 19.60 (95% CI 14.98–24.22) in the intervention group and 14.93 (95% CI 9.60–20.26) in the control group. At 3-months following post-test, the adjusted means of the SCAS were 17.48 (95% CI 13.07–21.88) and 14.63 (95% CI 9.55–19.72), respectively (Table 2 and Fig. 2). In primary analysis, the group difference from baseline SCAS scores by the MMRM analysis at the post-test were − 5.321 (95% CI − 10.12 to − 0.523, *p* = 0.030) and at the 3-month FU were − 7.104 (95% CI − 11.90 to − 2.306, *p* = 0.004).

**Table 2:** Estimated values and changes from baseline at each visit in SCAS and SDQ by MMRM

Score	Visit	IG (n = 40) Estimated mean (95% CI)	CG (n = 30) Estimated mean (95% CI)	Between group difference for baseline change	p value
SCAS	Pre	26.42 (21.74–31.11)	16.23 (10.83–21.64)	NA	
	Post	19.60 (14.98–24.22)	14.93 (9.60–20.26)	− 5.321 (− 10.12 to − 0.523)	0.030
	FU	17.48 (13.07–21.88)	14.63 (9.55–19.72)	− 7.104 (− 11.90 to − 2.306)	0.004
SDQ	Pre	13.10 (11.63–14.58)	9.53 (7.85–11.22)	NA	
	Post	11.39 (10.00–12.77)	9.53 (7.96–11.12)	− 1.975 (− 3.989 to 0.038)	0.054
	FU	11.51 (10.11–12.91)	10.87 (9.27–12.46)	− 3.284 (− 5.297 to − 1.270)	0.002

*SCAS* Spence Children’s Anxiety Scale, *SDQ* Strengths and Difficulties Questionnaire, *IG* Intervention Group, *CG* Control Group, *FU* follow-up, *NA* not available

**Fig. 3:**
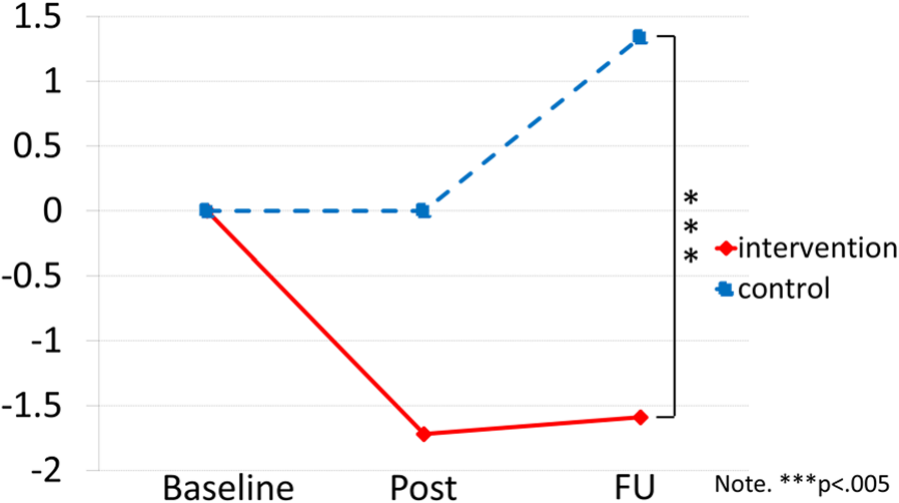
Mean total SDQ scores in each group during the study. It displays the average SDQ (total difficulties) scores of the intervention group and the control group for each time point

Showing the same pattern, the adjusted means of the SDQ were 11.39 (95% CI 10.00–12.77) in the intervention group and 9.53 (95% CI 7.85–11.22) in the control group. At 3-months following the post-test, the adjusted means of the SDQ were 11.51 (95% CI 10.11–12.91) and 10.87 (95% CI 9.27–12.46), respectively (Table 2 and Fig. 3). The group difference from baseline SDQ scores by MMRM analysis at the post-test were − 1.975 (95% CI − 3.989 to 0.038, *p* = 0.054) and at the 3-month FU were − 3.284 (95% CI −5.297 to − 1.270, *p* = 0.002).

**Fig. 4:**
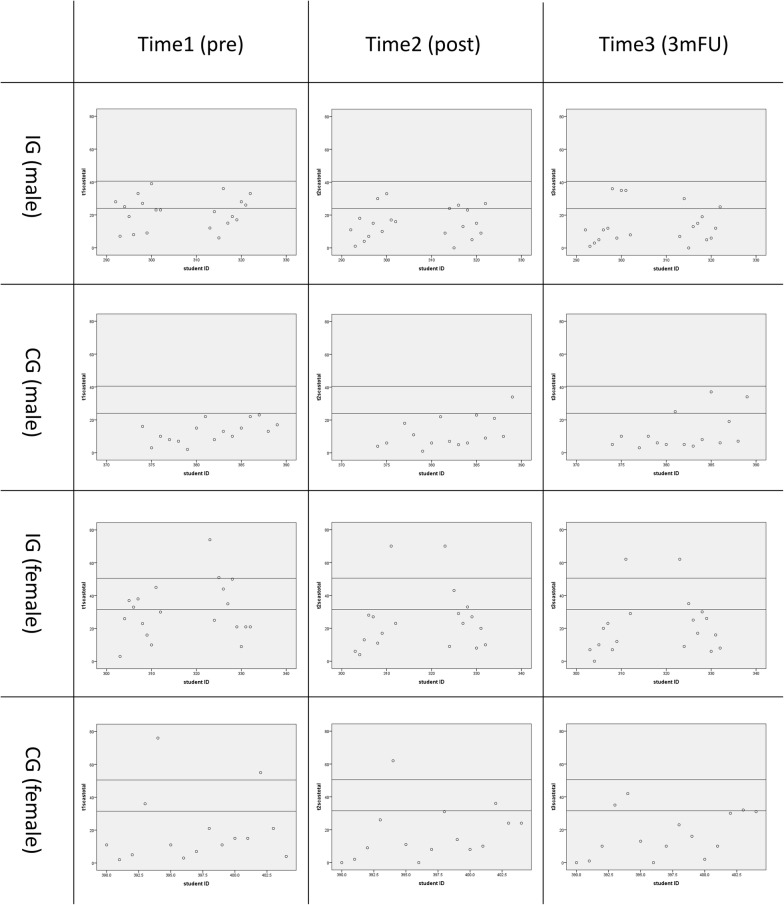
Scatter plot chart of SCAS score. It displays the scatter plot chart of the SCAS score of each child in the intervention and control group

To visually confirm the score change of each child in the intervention and control group, scatter plot charts were developed using the SCAS at pre-test, post-test, and FU, assigning scores of each group by sex vertically and children’s ID horizontally (Fig. 4).

In the plot chart, a baseline T-score of 50 (male = 24, female = 31–32) and T-score of 60 (male = 40–41, female = 50–51), based on the SCAS web site, were drawn as a standard line, and changes in the number of children above a T-score of 50 were counted (Table 3). The results indicated that the number of children with T-scores above 50 were reduced to about half in the intervention group, while the number stayed the same or increased in the control group.

**Table 3:** Change in child count in excess of T-score 50 (60)

	Pre	Post	FU
IG (male)	9 (0)	5 (0)	5 (0)
CG (male)	0 (0)	1 (0)	3 (0)
IG (female)	9 (2)	4 (2)	3 (2)
CG (female)	3 (2)	2 (1)	3 (0)

*IG* Intervention Group, *CG* Control Group, *FU* follow-up

## Discussion

The purpose of this study was to verify the feasibility and effectiveness of the CBT based ‘Journey of the Brave’ programme with a universal approach in a Japanese school class. The results of this study indicated that the SCAS and SDQ scores of the intervention group children were significantly reduced compared with the control group children. Therefore, the feasibility and effectiveness of this programme using a universal approach were supported in line with the previous pilot trial [32].

Currently, several programmes for the prevention of depression and anxiety are being developed based on the universal approach in school classes; however, it has been pointed out that although the effectiveness of anxiety prevention programmes has been proven to a degree, the effectiveness of depression prevention programmes is questionable. For example, Werner-Seidler conducted a systematic review in 2017 on school-based depression and anxiety prevention programmes for young people and reported that universal depression prevention programs had smaller effect sizes at post-test relative to targeted programmes. On the other hand, for anxiety, effect sizes were comparable for universal and targeted programmes [39]. The ‘Journey of the Brave’ programme was designed as a universal approach focusing on children’s anxiety problems based on a CBT model for the treatment of children’s anxiety disorders, and the positive results demonstrated in this study are in line with the result of Werner-Seidler’s study.

There are three major differences of this study compared with the aforementioned pilot study. The first is the reduction in frequency of programme sessions from once a week in the pilot study to twice a month in the current study. Usually, CBT treatment is conducted at a pace of once a week, and the pilot study adhered to this common interval. However, the class time of Japanese elementary and junior high schools is already fully occupied by the government-controlled curriculum and it was practically impossible to keep up with the originally planned pace. For this reason, we had to extend the interval between sessions to accommodate the request of the school. Initially we were concerned about a potential negative impact on children’s learning, but this study did not find noticeable negative effects; rather, significant reduction in the anxiety scores of the intervention group children was found. Thus, in implementing a CBT programme as a school based preventive education initiative, flexible session scheduling might be applicable according to the situation of each school, as opposed to strictly following the once a week guideline. Moreover, this extended period may enable children enough learning time to complete the homework, apply what was learned to their behaviour change, and consolidate the programme content.

The second major difference was that the programme was implemented in a universal setting in a school class. Our pilot study was originally designed as a universal programme, but could be suitable as a targeted level programme because we had to use only a limited number of recruited children from the community as participants. The effect size of this study could be smaller because targeted level studies tend to result in higher effect sizes [4]. However, the SCAS score of the intervention group was significantly reduced in this study, whereas the pilot study failed to find clear differences. Therefore, the effectiveness and feasibility of our ‘Journey of the Brave’ programme as a universal approach was verified. In addition, a scatter plot chart comparing intervention and control group children by sex was conducted. The result confirmed the reduction of children’s anxiety in the intervention group with T-scores above 50 from the post program period to 3-months after. A preventive programme basically targets healthy children; therefore, the anxiety scores of children whose initial score was low from the beginning did not require further reduction and are likely to show only a small reduction. However, at the same time, there are a limited number of children with high anxiety scores in each school and they are regarded as the high-risk group. Thus, children with T scores above 50 were treated as the high-risk group in this study, and their anxiety score changes were closely monitored. The results indicated that the number of children with T scores above 50 was reduced in both males and females after the programme intervention. This result suggests that this programme could possibly serve a role in early intervention for high-risk children.

The third difference is the usage of the SDQ as a secondary outcome measure to evaluate children’s behavioural problems. Considering the focus of the program, the SDQ score might demonstrate no positive results even if the SCAS score reduced significantly. However, the results of this study indicated equally positive outcomes on the SDQ compared with the control condition. The results indicate that the improvement in the children’s anxiety problems may have a positive influence on their behavioural problems.

In other countries, several evidence-based programmes addressing prevention of children’s anxiety are being developed and implemented in school classes with the assistance of local authorities and/or the national government. On the contrary, in Japan, although the necessity of prevention and measures to address children’s mental health problems are recognized, almost no time is dedicated to this issue in the school curriculum and there are limited class activities based on scientific evidence of psychosocial interventions. Thus, in a practical sense, it is up to each teacher to deal with mental health problems of children. With these considerations, we believe that it is quite a meaningful first step to show a new direction in children’s mental health problem prevention in Japan by demonstrating the feasibility and effectiveness of a universal prevention programme for anxiety symptoms in school classes as part of the regular session.

### Limitations and future prospects

We acknowledge several limitations in this study.

Firstly, a random assignment method was not applied to both intervention and control groups. When pre-programme scores are compared, the intervention group children showed significantly higher scores on both the SCAS and SDQ. While the average SCAS score as the primary outcome is reported to be 23.50 in Japanese children aged 7–11 [36], intervention group children in this study had an average score that was higher by 3 points and the control group children’s score was 7 points lower. This reflected the same tendency as the pilot study [34]. The reason for this imbalance is not clear, but it surely resulted from not using a random assignment method. Because of the utilization of the MMRM method to analyse the change of each score from baseline, results are displayed without considering the intergroup difference; to present the results in a more stringent manner, it would be better to randomize the sample before the intervention and start the statistical analysis from an equalized baseline.

It is reported that in the evaluation of prevention programme effectiveness, many studies have certain shortcomings in their research design and it is difficult to confirm that enough positive evidence has been demonstrated [40]. In this study, a quasi-experimental design was applied to judge the feasibility of implementing this programme at a universal level; to confirm its effectiveness, it is necessary to increase the number of participating schools to 10–20 and apply a cluster randomization method.

The second limitation of this study is the fact that this programme was facilitated by the first author herself who developed this programme and one of the authors who had knowledge of educational psychology. ‘Journey of the Brave’ is a programme developed for universal level execution in Japanese schools conducted by the classroom teacher. However, a preceding study pointed out that more positive results are likely to occur when it is facilitated by specialists [27, 28]. On the other hand, there are reports of studies with no significant differences in effectiveness, whether they were teacher-led or health care specialist-led [30]. With these considerations, to establish and spread this program as evidence-based, it would be necessary to verify its effectiveness even if the programme is facilitated by the class teacher. In addition, a thorough consideration of quality assurance such as holding a facilitator seminar is required at the stage of making this programme widespread so that the understanding of CBT theory is deepened.

The third limitation relates to the measurement scales. Our programme used the SCAS and SDQ, which essentially are symptom scales to measure anxiety score improvement. However, the purpose of ‘Journey of the Brave’ is to prevent anxiety related problems and in verifying programme effectiveness, it is essential not only to compare pre- and post-programme scores, but to observe the process of improvement of children and conduct longitudinal studies such as those addressing the prevalence of anxiety disorders and the number of children with school refusal problems.

To evaluate the effectiveness of this preventive programme, it would be advisable not only to apply an RCT design, but also to continuously confirm its long-term effectiveness with longitudinal studies in Japan.

## Conclusions

The results of this study confirmed the feasibility and effectiveness of a CBT based anxiety prevention programme, ‘Journey of the Brave’, when conducted at a Japanese school. In this study, we believe it is quite important that this preventive CBT program was implemented in actual school classes as a part of regular sessions. However, at the same time, there are several limitations in the study design and it is necessary to apply cluster randomization methods and to verify its preventive effects in a vertically integrated manner in the future.

## Abbreviations

CBT: cognitive behaviour therapy
CI: confidence interval
FU: follow-up
SCAS: Spence Children’s Anxiety Scale
SDQ: Strengths and Difficulties Questionnaire
ITT: intention to treat
MMRM: mixed-effect model for repeated measures
